# Conceptual disorganization and redistribution of resting-state cortical hubs in untreated first-episode psychosis: A 7T study

**DOI:** 10.1038/s41537-020-00130-3

**Published:** 2021-01-26

**Authors:** Avyarthana Dey, Kara Dempster, Michael MacKinley, Peter Jeon, Tushar Das, Ali Khan, Joe Gati, Lena Palaniyappan

**Affiliations:** 1 Robarts Research Institute, London, ON Canada; 2 Department of Psychiatry, University of Western Ontario, London, ON Canada; 3 Lawson Health Research Institute, London, ON Canada; 4 Department of Medical Biophysics, University of Western Ontario, London, ON Canada; 5 The Brain and Mind Institute, University of Western Ontario, London, ON Canada; 6 Present Address: Department of Psychiatry, Dalhousie University, Halifax, NS Canada

**Keywords:** Neuroscience, Schizophrenia

## Abstract

Network-level dysconnectivity has been studied in positive and negative symptoms of schizophrenia. Conceptual disorganization (CD) is a symptom subtype that predicts impaired real-world functioning in psychosis. Systematic reviews have reported aberrant connectivity in formal thought disorder, a construct related to CD. However, no studies have investigated whole-brain functional correlates of CD in psychosis. We sought to investigate brain regions explaining the severity of CD in patients with first-episode psychosis (FEPs) compared with healthy controls (HCs). We computed whole-brain binarized degree centrality maps of 31 FEPs, 25 HCs, and characterized the patterns of network connectivity in the 2 groups. In FEPs, we related these findings to the severity of CD. We also studied the effect of positive and negative symptoms on altered network connectivity. Compared to HCs, reduced centrality of a right superior temporal gyrus (rSTG) cluster was observed in the FEPs. In patients exhibiting high CD, increased centrality of a medial superior parietal (mSPL) cluster was observed, compared to patients exhibiting low CD. This cluster was strongly correlated with CD scores but not with other symptom scores. Our observations are congruent with previous findings of reduced but not increased centrality. We observed increased centrality of mSPL suggesting that cortical reorganization occurs to provide alternate routes for information transfer. These findings provide insight into the underlying neural processes mediating the presentation of symptoms in untreated FEP. Longitudinal tracking of the symptom course will be useful to assess the mechanisms underlying these compensatory changes.

## Introduction

Disorganization is one of the three distinct syndromes of schizophrenia^1–4^, defined collectively as the impairment of the form of thought processes (conceptual disorganization (CD)) and bizarre actions (behavioral disorganization). CD, in particular, is comprised of difficulties in the goal-directed sequencing of thoughts that manifest as circumstantial, illogical, or tangential speech or weakened goal of thinking (loose associations)^5^. CD has been shown to be tightly linked to real-world functioning^6–8^. CD is also referred to as “formal thought disorder (FTD)”, though the latter is often measured using specific instruments that focus on testing aspects of speech rather than the clinical interpretation of disorganization. While the construct of FTD encompasses both positive (e.g., illogical, tangential, and loosened speech) as well as negative (e.g., poverty of speech and reduced content of speech) FTD, the clinical construct of CD refers only to positive aspects of FTD.

CD is seen in both acute and chronic stages of established psychosis (schizophrenia)^9^, with some indication that the pathophysiology of the persistent CD may be distinct from the acute CD seen during an active psychotic episode^10^. For example, the acute CD resolves in many patients over time^11–14^, and often covaries with the severity of positive symptoms such as delusions and hallucinations, while the CD seen in chronic stages often emerges as a distinct subtype, with more negative features of FTD being noted^15^. The persistence of CD despite treatment in longitudinal cohorts has also led to the suggestion that existing treatments are not fully effective in alleviating the degree of disorganization in patients with psychosis^12,16,17^.

A large body of neuroimaging literature has attempted to parse the brain regions implicated in the thought disorders of schizophrenia^18^, with several comprehensive systematic reviews^15,18,19^ providing an excellent overview of the reported brain correlates of CD (more specifically, FTD). These reviews indicate that a large number of studies have focused explicitly on aberrations in language processing brain regions (especially superior temporal gyrus (STG)) as the major structural and functional underpinnings of FTD^20,21^. Nevertheless, null findings, as well as findings that implicate brain regions that are not considered to be a part of the language network, are also reported^20^. A notable lack of studies directly relating network-level functional connectivity to disorganization has been highlighted^19^.

A limited number of resting-state functional magnetic resonance imaging (rs-fMRI) studies have investigated the network level dysconnectivity underlying disorganization. These studies report reduced connectivity between the thalamus and postcentral gyrus^22^, between the frontoparietal and cerebellar networks^23^, between the dorsolateral prefrontal cortex (DLPFC) and insula, Wernicke’s area, sensorimotor area, and frontal pole, and increased connectivity between DLPFC and premotor cortex^24^ in relation to pronounced disorganization or FTD. Taken together, dysconnectivity of several brain regions outside of the traditional language network appears to contribute to the severity of disorganization. Several studies have also failed to find a relationship between severity of disorganization and functional connectivity^18,19,25,26^. All of these studies have been conducted on established cases of schizophrenia, with patients recruited during a stable medicated phase of the illness. Thus, the neural basis of acute disorganization seen during first-episode psychosis (FEP) is still unclear. Furthermore, to our knowledge, there have been no studies to date that investigate voxel-by-voxel whole-brain connectivity in relation to disorganization.

In the current study, we studied acute CD from a sample of 38 untreated patients with FEP and 31 age-matched healthy controls. We acquired rs-fMRI scans from an ultra-high field 7-tesla MRI scanner. Without making any a priori assumptions about the brain regions implicated in acute disorganization, we first undertook a whole-brain, voxel-wise search to locate brain regions showing aberrant connectivity in relation to the presence of psychosis as well as the severity of disorganization. To establish the specificity of our findings to the symptom of CD, we studied the effect of other positive and negative symptoms of psychosis in relation to the observed results.

## Results

### Demographics and clinical characteristics

All patients were acutely psychotic, minimally treated, and had significant social and occupational dysfunction at the time of scanning. The average duration of illness (including prodrome) was 302 days (SD = 495 days), with the mean number of days of exposure to antipsychotic medication being <4 days at the time of scanning.

The demographic and clinical characteristics of the sample are presented in Table 1 (and Supplementary Table 2 for the combined patients groups with high and low CD). The two patient groups did not differ from HCs in terms of age, gender, and socio-economic status. The two patient groups did not differ among themselves in terms of age, gender, socio-economic status, duration of untreated illness in days, exposure to antipsychotic medication in days, a total daily dose of antipsychotic medication, and the number of patients with schizophrenia in either group (diagnosed 6 months after the initial presentation). Across the three groups, we observed a significant difference for the SOFAS and DSST (mean) scores, confirmed by an ANOVA. We use binarised degree centrality (bDC) as the primary measure of whole-brain connectivity (see Methods).

**Table 1:** Demographic and clinical characteristics of the sample.

Measure	High P2	Low P2	Healthy controls	Group statistics
Number of subjects	12	26	31	–
Age (years)	21 (2.80)	23.15 (5.04)	21.61 (3.26)	*F* (2,66) = 1.609; *p* = 0.208
Gender (M/F)^^^	11 / 1	21 / 5	19 / 12	*χ*^2^ (2, *N* = 69) = 5.157; *p* = 0.075
Exposure to antipsychotic medication (days)	2.57 (3.55)	5.53 (4.48)	–	*U* = 36.500, *p* = 0.136
Duration of untreated illness (days)	207 (186)	343 (579)	–	*U* = 113.000, *p* = 0.938
Total DDD of antipsychotics	1.04 (1.79)	2.91 (2.95)	–	*U* = 36.500, *p* = 0.136
Diagnosis (SCZ/other psychoses)^^^	8/4	21/5	–	*χ*^2^ (1, *N* = 38) = 0.903; *p* = 0.342
NSSEC	3.67 (1.22)	3.47 (1.30)	3.20 (1.42)	*F* (2,55) = 0.504; *p* = 0.607
Conceptual disorganization (P2)^†^	4.5 (0.67)	1.73 (0.92)	–	*U* = 0.000, *p* < 0.001
Lack of spontaneity and flow of conversation (N6)^†^	1.92 (0.996)	2.04 (1.31)	–	*U* = 154.500, *p* = 0.959
Total positive component (P1 + P3 + G9)	14.10 (2.02)	13.00 (2.56)	–	*U* = 123.500, *p* = 0.303
Total negative component (N1 + N4)	5.80 (3.26)	5.61 (3.22)	–	*U* = 147.000, *p* = 0.774
Speech rate and amount (YMRS 6)^†^	0.75 (1.49)	0 (0)	–	*U* = 87.500, *p* < 0.001
Language/thought disorder (YMRS 7)^†^	2.88 (1.81)	0.75 (1.41)	–	*U* = 40.000, *p* < 0.001
SOFAS	37.50 (8.81)	41.83 (13.69)	79.96 (4.77)	*F* (2,57) = 109.17; *p* < 0.001*
DSST (mean)	53.00 (17.10)	53.27 (12.90)	68.63 (10.93)	*F* (2,66) = 12.259; *p* < 0.001*

*DDD* defined daily dose, *M* mean, *SD* standard deviation, *SOFAS* social occupational functioning assessment scale, *NSSEC* National Statistics Socio-Economic Classification score, *DSST* modified digit symbol substitution test; schizoaffective disorder and schizophreniform disorder are referred to as “other psychoses”.^^^ chi-square test, ^†^Mann–Whitney *U* test, *high P2 = Low P2 < HC; *DDD* defined daily dose of antipsychotics, *high P2* patients with high CD, low P2 patients with low CD, *YMRS* Young’s mania rating scale.

### Effect of psychosis on binarized degree centrality (bDC)

**Fig. 1:**
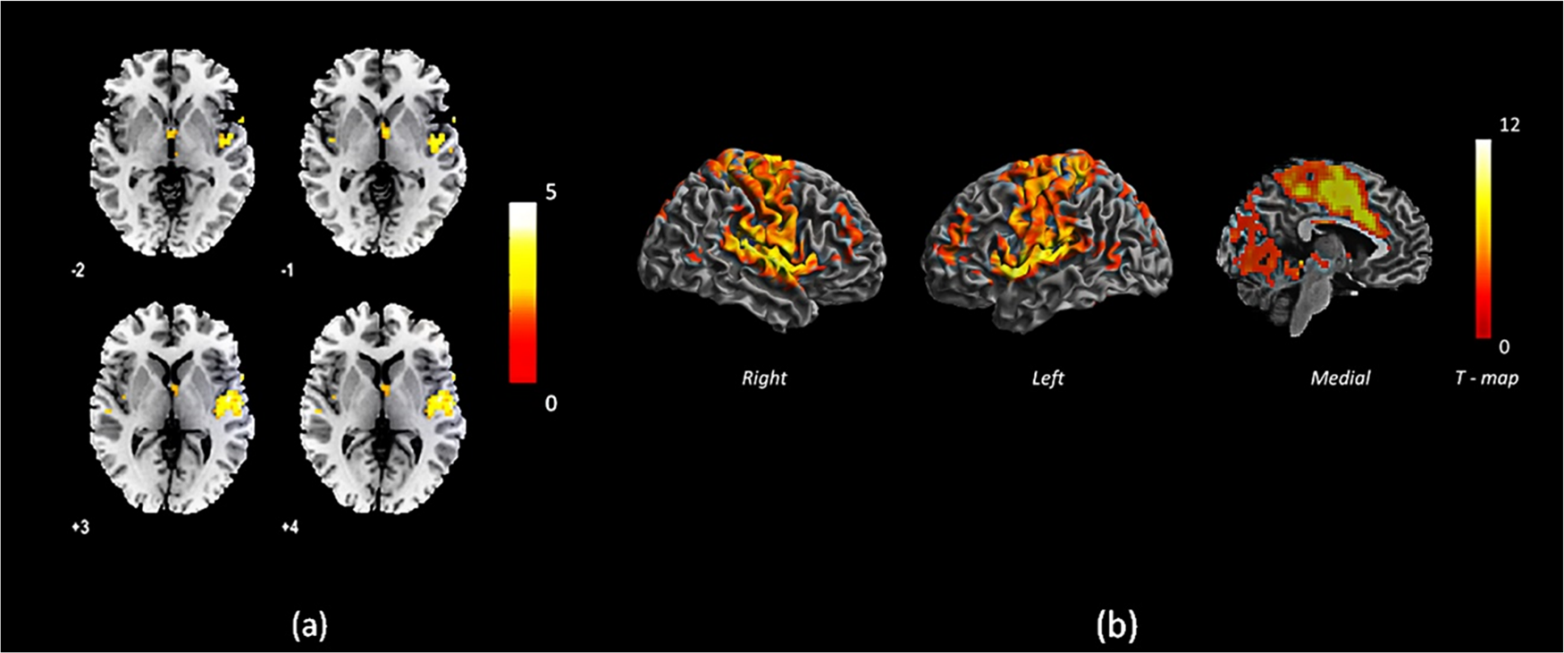
Cluster with reduced centrality and its functional connectivity in FEP compared to healthy control subjects. **a** Brain regions showing reduced centrality in the FEP (*n* = 38) group compared to HCs (*n* = 31), **b** Functional connectivity of the STG/insula cluster to other brain areas for subjects in the FEP group (*n* = 38). The colour bars indicate *T*-values.

Two sample *t* tests of binarized degree centrality (bDC) between the two groups (FEP, HC) revealed a significant reduction in the centrality of the right STG, right insula, and right Heschl’s gyrus in patients with FEP compared to HCs. The group differences are shown in Table 2 and Fig. 1a. No significant increases in centrality were observed in the patient group compared to HCs.

**Table 2:** Brain regions showing reduced centrality in the FEP (*n* = 38) group compared to HCs (*n* = 31).

Brain regions included in cluster (AAL labels)	Cluster size	Peak MNI coordinates			*T*-value	*p* Value (cFWE corrected)
		*x*	*y*	*z*		
FEP < HC						
*STG*/*insula cluster*	148	48	−9	3	5.00	0.001
Temporal_Sup_R	75					
Insula_R	46					
Heschl_R	22					
FEP > HC						
Not significant						

*cFWE* cluster FWE, MNI Montreal Neurological Institute, *FEP* first episode psychosis, *HC* healthy controls, *AAL* automated anatomical labeling.

In seed-based FC, within the entire FEP group, we noted that the right STG/insula cluster—a cluster showing significant reductions in centrality—showed significant functional connectivity with a set of distributed brain regions, including bilateral insula, middle cingulate region, SMA, as well as clusters in bilateral thalamus, bilateral calcarine, and lingual regions extending to the cerebellum (lobules 4–6), and two clusters located at the left and right middle frontal gyrus. This indicates that the right STG/insula cluster belongs to a distributed network of other highly connected hub regions of the salience network as well as the executive network (Table 3 and Fig. 1b). [Also see Supplementary discussion 1 for the meta-analytic functional connectivity data from Neurosynth database]. We did not find any significant differences in the functional connectivity between patients and control subjects on a two-sample *t* test, indicating that the reduced centrality of the right STG/insula cluster in psychosis is not driven by any specific network-level reductions in connectivity.

**Table 3:** Functional connectivity of the STG/insula cluster to other brain areas for subjects in the FEP group (*n* = 38).

Brain regions included in cluster (AAL labels)	Cluster size	Peak MNI coordinates			*T*-value	*p* Value (cFWE corrected)
		*x*	*y*	*z*		
*STG*/*insula cluster*	10,298	51	−6	3	13.23	<0.001
Postcentral_R/L	739					
Temporal_Sup_R/L	597					
Insula_R/L	376					
Precentral_R/L	563					
Paracentral_Lobule_R/L	241					
Supp_Motor_Area_R/L	384					
*Thalamus cluster*	307	−12	−21	3	9.72	<0.001
*Cerebellum cluster*	447	−33	−24	3	8.02	<0.001
Calcarine_R/L	333					
Cerebelum_4_5_R/L	232					
Cerebelum_6_R/L	210					
Lingual_R/L	202					
*Left middle frontal gyrus cluster*	344	−30	33	33	8.41	<0.001
*Right middle frontal gyrus cluster*	194	39	51	15	7.59	<0.001

*cFWE* cluster FWE, *MNI* Montreal Neurological Institute, *AAL* automated anatomical labeling.

### Effect of CD on bDC

**Fig. 2:**
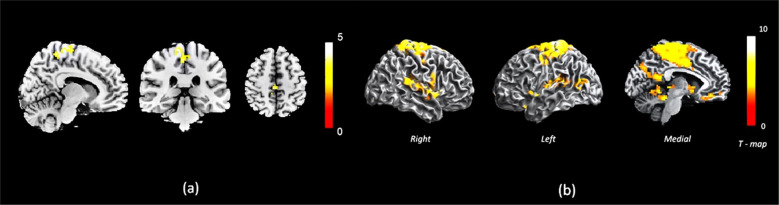
Cluster with reduced centrality and its functional connectivity in FEP with high vs. low conceptual disorganisation. **a** Brain regions showing reduced centrality in the High P2 group (*n* = 12) compared to the Low P2 group (*n* = 26). **b** Functional connectivity of the medial superior parietal lobule cluster to other brain areas in the FEP group (*n* = 38). The colour bars indicate *T*-values.

In patients with high P2 (item P2 of Positive and Negative Syndrome Scale PANSS) compared to low P2, we observed a significant increase in bDC of a medial superior parietal cluster, comprising of paracentral lobule and precuneus regions. Results are shown in Table 4 and Fig. 2a. The low P2 group did not differ from the healthy controls in the bDC of this cluster (*t*[55] = 1.6, *p* = 0.11) while the high P2 group had significantly higher bDC than healthy controls (*t*[36] = 4.94, *p* < 0.001) (mean (SD) in controls = 0.35 (0.33); low P2 = 0.22 (0.23); high P2 = 0.68 (0.33); see Supplementary Result 2 for more details).

**Table 4:** Brain regions showing reduced centrality in the high P2 group (*n* = 12) compared to the low P2 group (*n* = 26).

Brain regions included in cluster (AAL labels)	Cluster size	Peak MNI coordinates			*T*-value	*p* Value (cFWE corrected)
		*x*	*y*	*z*		
*High P2* > *Low P2*						
*Medial superior parietal lobule cluster*	88	−12	−30	69	4.93	<0.001
Paracentral_Lobule_R/L	59					
Precuneus_R/L	17					

*cFWE* cluster FWE, *MNI* Montreal Neurological Institute, *AAL* automated anatomical labeling.

In patients with FEP as a group, the medial superior parietal cluster showed significant functional connectivity with a cluster comprising of the post/precentral regions, middle cingulate cortex and SMA, as well as bilateral STG and right middle temporal gyrus. This indicates that the region showing increased centrality among patients with a high degree of disorganization is functionally connected to sensorimotor (post/precentral regions), motor planning and control (middle cingulate cortex and SMA) regions and to a limited extent, to language processing regions (bilateral STG and right middle temporal gyrus) in patients with FEP (Table 5 and Fig. 2b).

**Table 5:** Functional connectivity of the medial superior parietal lobule cluster to other brain areas in the FEP group (*n* = 38).

Brain regions included in cluster (AAL labels)	Cluster size	Peak MNI coordinates			*T*-value	*p* Value (cFWE corrected)
		*x*	*y*	*z*		
*Precuneus/SMA cluster*	4838	−12	−27	75	9.45	<0.001
Postcentral_R/L	351					
Precuneus_R/L	342					
Cingulum_Mid_R/L	279					
Precentral_R/L	261					
Paracentral_Lobule_R/L	255					
Supp_Motor_Area_R/L	233					
*Gyrus rectus cluster*	195	9	30	−21	7.94	<0.001
Rectus_R/L	55					
Frontal_Med_Orb_R	50					
*Sup. Temporal gyrus cluster*	466	54	−18	6	8.39	<0.001
Temporal_Sup_R/L	247					
*Middle temporal gyrus cluster*	130	51	−66	9	7.07	<0.001
Temporal_Mid_R	121					

*cFWE* cluster FWE, *MNI* Montreal Neurological Institute, *AAL* automated anatomical labeling.

### The specificity of findings to disorganization

To test the specificity of the relationship between CD and increased bDC of medial SPL cluster, we related the eigenvariate of bDC from this cluster for each patient (dependent variable) to all of the individual symptom scores as well as SOFAS and DSST (P1–P3, N1, N4, N6, G5, G9, SOFAS, DSST score: independent variables) in a univariate GLM model. Only P2 emerged as a significant predictor in this model (*t* = 2.24, *p* = 0.035; all other predictors *p* = 0.09-0.84), indicating the specificity of the relationship between CD and medial SPL centrality. Mean FD, age, and clinical global impression (CGI) severity score had no significant correlations with mSPL bDC (Spearman’s correlation, uncorrected *p* ranging from 0.17 to 0.58). Furthermore, there were no systematic differences in the distributions of clinical and demographic variables (other than P2 scores) between the two groups of patients. There were no differences in head motion (FD) between the 2 patient groups (*t* = 0.03, *p* = 0.98). None of the reported clusters from group contrasts systematically related to FD (mSPL cluster in patients *r* = −0.04, *p* = 0.82; in controls *r* = 0.08, *p* = 0.66; STG/INS cluster in patients *r* = 0.12, *p* = 0.46; in controls *r* = −0.04, *p* = 0.82).

We observed a significant relationship between P2 scores and bDC of the medial superior parietal cluster (Spearman’s rho = 0.42, *p* = 0.009), confirming that the observed results are not influenced by the cut-off used to identify highly disorganized FEP subjects.

We did not find any significant differences in the functional connectivity between the high P2 and low P2 groups on a two-sample *t* test, indicating that the increase in the centrality of the paracentral cluster in the high P2 group is not driven by any localized aberrations in connectivity.

## Discussion

To our knowledge, this is the first voxel-wise whole-brain study investigating the neural basis of acute CD. In an acutely psychotic sample representative of the minimally treated patients with FEP, we report the following three major findings using ultra-high-field 7 T resting-state fMRI data: (1) Patients with acute FEP show reduced centrality of the right superior temporal cortex, a region that is functionally coupled to many other established cortical hubs (insula, midcingulate cortex, thalamus, and DLPFC), (2) highly conceptually disorganized patients, compared to those with lower levels of disorganization, demonstrate a significant increase in the centrality of the medial superior parietal region that is functionally coupled to sensorimotor regions (3) This pattern of increased centrality observed in a task-free resting state among the acutely symptomatic FEP patients is specific to the symptom of CD, not influenced by other positive/negative symptoms or degree of reduced functioning among patients.

Our observation of reduced centrality affecting STG and insula is consistent with our prior work in a medicated sample of patients with schizophrenia^27^. In that previous study, we also observed increased centrality in the hippocampus, thalamus, inferior temporal, and occipital regions in patients with schizophrenia compared to healthy controls. In the current acutely psychotic, early stage sample, the lack of any increased centrality in FEP indicates that the emergence of such peripheral hubs in the visual cortex and medial/inferior temporal regions may be a feature of chronicity or treatment effect rather than being a feature of psychosis per se.

We observed an association between increased centrality of paracentral/medial superior parietal cluster, a region that is not conventionally regarded as a functional hub, and CD. Interestingly, this cluster was functionally connected to the superior and middle temporal regions as well as regions comprising the sensorimotor network^28^. Assuming that the pathophysiology of psychosis is a generalized dysconnectivity that has a preponderance to affect regions such as STG due to their higher centrality, one would not expect to see an increased centrality of peripheral, sensory nodes in a subgroup of patients. In this context, our results reaffirm the notion that distinct pathophysiological processes are likely to underlie the myriad of symptoms seen in psychosis.

The observed reduction in centrality across core hubs with a smaller magnitude of increase in centrality in other regions is also consistent with many prior observations of reduced degree centrality^29–31^, though Tang et al.^32^ reported no centrality reductions in schizophrenia. Tang and colleagues studied a relatively younger age group of subjects with >50% medicated for an unspecified amount of time; and considered only positive values of functional connectivity to quantify centrality. These methodological and sample related variations may explain the differences between our results and the report of Tang and colleagues.

Our observation does not necessarily negate a role for language networks in thought disorders; instead, it provides empirical support for the role of system-level dysconnectivity at resting-state as a determinant of the severity of FTD. Our results are not directly comparable to the results obtained from studies on patients with persistent thought disorder^33,34^. Ketamine challenge which recapitulates acute thought disorder has been reported to increase functional (BOLD) activity in the same medial sensory association cluster where we observed a higher level of bDC among the more disorganized patient group^35^. A white fiber bundle recognized to participate in language production^36^ connects the sensory association cortex to the auditory association cortex^37^. Our functional connectivity analysis also revealed a significant relationship between superior and middle temporal areas and the medial cluster located on the sensory association area in patients with FEP. It is possible that the primary “downstream” interference caused by an emergent hub in the sensory association cortex is mediated via the language network.

In so far as functional connectivity reflects the ability to transmit information across distant brain regions, it is possible that the emergence of peripheral (sensory) hubs could be a compensatory mechanism to preserve brain function. This may result in a re-routing of information transmitted via functional brain networks^38^. Such rerouting could result in an aberrant shunting of associative processes (in line with Wernicke’s notion of sejunction^39^), resulting in disorganized speech. Emerging observations from lesional studies indicate that flexible redistribution. within and across networks is a ubiquitous feature of robust cognitive networks^40^. In this regard, we use the term redistribution to signify 2 aspects of changes in the brain that follows a pathological insult: (1) A change in the relative contribution of different brain regions to a specific cognitive function. (2) The preservation of global topological balance in the connectome through more localized changes in connectivity (as seen during ontogeny). Reviewing this literature^40^, Hartwigsen observes that the efficiency of functional compensation is most efficient if regions from the same domain-specific network (in our case, the language network) are recruited instead of regions that are not initially engaged in a specific task^41^. In this regard, the CD can be considered to be the result of the emergence of peripheral hubs with no domain-specific role in language processing.

While most previous studies that recruited patients with persistent disorganization in later stages of illness implicated a role for the language processing regions, we note STG centrality to be reduced in both high and low P2 groups—albeit to different extents (Supplementary Table 2). It is worth noting that the extant literature implicating language regions in thought disorder sought changes in language network a priori, rather than taking a discovery approach without task constraints^19,20^ could identify only two rs-fMRI studies investigating this issue, despite their exhaustive search. Interestingly, these few whole-brain studies have reported sensorimotor dysconnectivity in association with thought disorder. It is also important to note that STG changes seen in patients with psychosis are not specific to thought disorder^20^. For instance, a large body of structural and functional studies implicate STG in negative symptoms^42,43^, as well as auditory hallucinations^44,45^. Our findings support the notion that language network dysfunction is likely to be a common feature across the various symptomatic subgroups of psychosis, at least in the early, untreated stages of illness.

We assessed both a positive (P2 of PANSS) and a negative (N6 of PANSS) feature of FTD and further corroborated the presence of disorganization using complementary clinical definitions (YMRS scale items 6 and 7). Nevertheless, our results cannot be taken to represent the neural basis of FTD as such, as we lacked instruments that comprehensively quantify the various aspects of FTD (see Supplementary Note 1). We recommend multidimensional assessment in a future studies to capture the linguistic as well as clinical aspects of FTD, as they may not fully overlap^46^. We had disproportionately fewer female participants; caution is required when generalizing our results in this regard. The choice of our analytical procedures may also have influenced our results (See Supplementary Fig. 1).

To conclude, in the presence of reduced centrality of the superior temporal hub region, a relative increase in the centrality of sensory association regions is seen in highly disorganized patients with FEP. We postulate that an inefficient cortical information transfer resulting from the emergence of peripheral hubs underlies the pathogenesis of disorganization among patients with psychosis.

## Methods

### Participants

The sample consisted of 51 consecutive new referrals to the PEPP (Prevention and Early Intervention for Psychosis Program) at London Health Sciences Center, London, Ontario, Canada between April 2017 and July 2018. The PEPP program uses an assertive case-management model to provide assessment and treatment to individuals 16–39 years old experiencing FEP. All potential study participants provided written, informed consent prior to participation as per the approval provided by the Western University Health Sciences Research Ethics Board, London, Ontario.

Inclusion criteria for study participation were as follows: individuals experiencing a first episode of psychosis and having received antipsychotic treatment for less than 14 days in their lifetime. Both inpatients and outpatients were eligible to participate if they were able to provide informed consent and safely participate in the MRI protocol. All participants received a consensus diagnosis from three psychiatrists (L.P./K.D. and the primary treatment provider) after approximately 6 months on the basis of the best estimate procedure (as described in ref. ^47^) and the Structured Clinical Interview for DSM-5^48^. Following the 6-month consensus diagnosis, participants meeting criteria for bipolar disorder with psychotic features, a major depressive disorder with psychotic features, or suspected drug-induced psychoses were excluded from further analyses. Care was provided as usual to the study participants through their psychiatrist and other allied health members with the PEPP program. Antipsychotic medications were chosen by the treating psychiatrist in collaboration with the patient and/or their substitute decision-maker. Individuals were offered the option of treatment with a long-acting injectable at the earliest opportunity^49^ in accordance with current national guidelines for the treatment of FEP.

Recruitment of healthy control subjects (*n* = 31) was carried out through posters that advertised the opportunity to participate in a neuroimaging study to track the outcomes of FEP. Healthy control subjects had no personal history of mental illness and no family history of psychotic disorders. Group matching with the FEP cohort for age, gender, and parental socio-economic status was maintained.

Exclusion criteria for both the FEP and healthy control groups involved meeting criteria for a substance use disorder in the past year according to DSM-5 criteria^48^, having a history of a major head injury (leading to a significant period of unconsciousness or seizures), having a significant, uncontrolled medical illness, or having any contraindications to undergoing MRI.

### Clinical and cognitive assessments

The severity of symptoms in patients was assessed using the 8-item positive and negative syndrome scale (PANSS-8) which measures the severity of positive and negative symptoms. We also used the Social and Occupational Functioning Assessment Scale (SOFAS) to assess the overall level of functioning^50^. We also used a modified digit symbol substitution task (DSST) to quantify processing speed, a cognitive function that shows the most prominent reduction among patients with psychosis^51^. The written and oral items on the DSST were scored separately, and the mean scores from these items were used for assessment as in our prior study^52^.

Scores from the PANSS-8 scale were grouped into a positive component (P1, P3, and G9 measuring “delusions”, “hallucinations”, and “unusual thought content”, respectively), a negative component (N1, N4, and N6 measuring “blunted affect”, “passive/apathetic social withdrawal”, and “lack of spontaneity and flow of conversation”, respectively) and P2 measuring “CD”. These eight items were chosen as they are the most indicative of the achievement of clinical remission when patients receive treatment^53^ scores. The PANSS-8 was applied by one of the two research psychiatrists (L.P. or K.D.) on the same week of the MRI acquisition, prior to the patients receiving clinically adequate treatment (i.e., at first contact on presentation). In addition, we also quantified the severity of two domains of negative symptoms using the brief negative symptom scale (BNSS)^54^ and overall illness severity using CGI^55^.

Patients who scored 1, 2, or 3 points on individual PANSS items were categorized to have low severity of the specific symptom (e.g., low P2), compared to those who scored 4, 5, 6, or 7 (high P2). We chose this cut-off to distinguish those who have clinically severe vs. minimal symptoms recommended by the Remission Working Group^53^. This cut-off has also been recently employed to distinguish patients with or without FTD and language dysfunction^56^.

As different antipsychotics were prescribed, we used WHO’s algorithm for defined daily doses (DDD) for antipsychotic medications^57^ to obtain a common unit of exposure. DDD refers to a specific amount i.e., a ratio unit of the DDD whose absolute milligram units vary among the different medications.

See Supplementary Note 1 for a review on the influence of assessment procedures used in this study and Supplementary Fig. 1 for a frequency distribution of ratings for the PANSS-P2 scores across the sample.

### MRI data acquisition

All magnetic resonance (MR) images were acquired on a 7.0 T Siemens (Erlangen, Germany) Magnetom MR imaging (MRI) scanner using a 32-channel head coil at the Center for Functional and Metabolic Mapping (CFMM), Robarts Research Institute, Western University.

A T2*-weighted 2D gradient echo-planar imaging (EPI) sequence with 360 volumes [acquisition time = 6 min; repetition time (TR) = 1000 ms; echo time (TE) = 20 ms; flip angle = 30°; FOV (read, phase) = 208 mm, 100%; number of slices = 63; slice thickness = 2 mm] was collected. Subjects were instructed to lay still inside the scanner with their eyes open for the duration of the scan and not to think of anything in particular. Foam pads were used to minimize subject head motion, and headphones were used to reduce scanner noise. Dummy scans were acquired prior to the acquisition of resting-state functional volumes to allow for the stabilization of both the signal and magnetic field. High-resolution T1-weighted sequences were collected for co-registration with the EPI and had the following parameters: acquisition time = 9 min 38 s; TR = 6000 ms; TE = 2.83 ms; flip angles = 4°, 5°; FOV (read, phase) = 240 mm, 100%; number of slices = 63; slice thickness = 0.75 mm.

### Data preprocessing

**Fig. 3:**
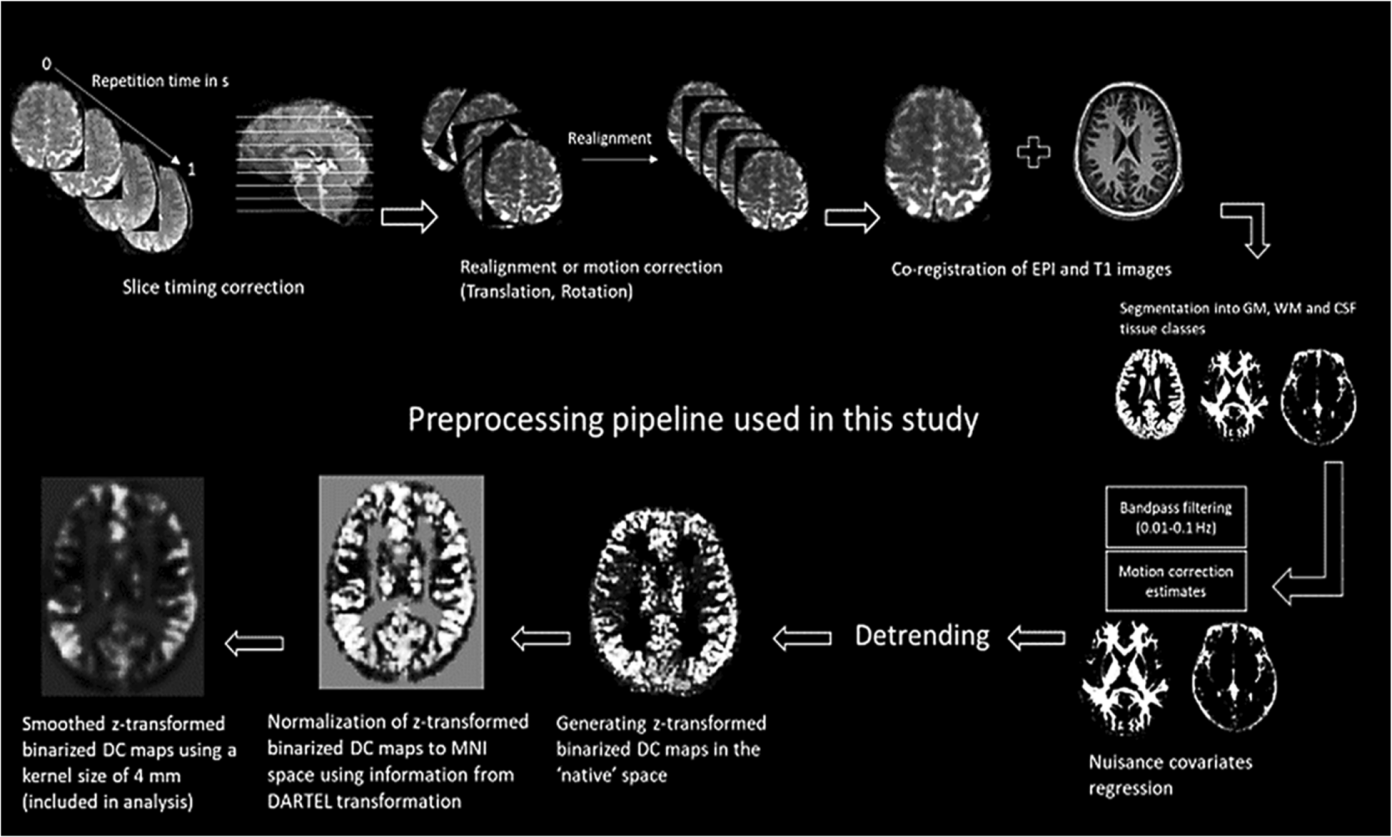
Steps of preprocessing performed in this study. GM grey matter, WM white matter, EPI echo-planar imaging, DARTEL diffeomorphic anatomical registration through exponentiated lie algebra, DC degree centrality.

All steps of data processing and analysis are displayed in Fig. 3. Preprocessing of rs-fMRI data was performed using the Data Processing Assistant for Resting-State fMRI Advanced toolbox^58^ ([http://rfmri.org/DPARSF), which is based on the Statistical Parametric Mapping software^59^ (http://www.fil.ion.ucl.ac.uk/spm) and the toolbox for Data Processing & Analysis of Brain Imaging^60^ (http://rfmri.org/DPABI). All 360 functional volumes (rs-fMRI) acquired from each subject were corrected for differences in slice acquisition times, following which images were spatially realigned to the mean image of the dataset to correct for small movements that occurred between scans. Individual T1-weighted structural images were co-registered to the mean of the realigned EPI images, followed by segmentation into gray matter, white matter, and cerebrospinal fluid^61^ tissue classes. The “Diffeomorphic Anatomical Registration using Exponentiated Lie algebra” or DARTEL toolbox^62^ used the information from the previous step to generate tissue class images which were rigidly transformed and in close alignment with SPM tissue probability maps.

Rs-fMRI signal measures have been shown to be highly sensitive to micro-scale head motions^63^. We derived the 24 motion parameters^64^ (6 head motion parameters [*t*], 6 head motion parameters at the previous time point [*t* − 1], and the squared values of these 12 measures). Participants with a head motion estimate of >3 mm (translation) and >3° (rotation) were excluded from the final sample. For the remaining participants, any variance in BOLD signal amplitude related to estimated head motion parameters was regressed out from the realigned data, before further processing.

To control for the effects of confounding factors, linear and quadratic trends in the data as well as the signals from the white matter and cerebrospinal fluid were removed from the data with linear regression^65^. We did not regress out the global signal, given its reported relationship to symptom severity in schizophrenia^66^. Following temporal filtering using a band-pass filter (0.01–0.1 Hz), linear detrending and standardization procedures were performed on the EPI images. The head motion “scrubbing” routine in DPARSFA was used to correct for framewise displacement (FD) motion artifacts using the “nearest neighbors” interpolation method (FD threshold of 0.5; interpolated 1-time point before and 2-time points after the flagged frame, in line with^67^). For each subject, we also estimated the mean FD in mm, averaged across a total of 360 acquisition time points to estimate the overall degree of motion^68,69^ for group comparisons.

We excluded 13 subjects due to motion during acquisition (*n* = 5), failure of quality check during preprocessing (*n* = 5), experiencing anxiety inside the scanner (*n* = 2), not resting during the scan (*n* = 1) (13 FEPs, 0 HCs). The excluded subjects did not differ from the patients who were included in the final sample in terms of age, gender and PANSS-8 total, DSST, NSSEC, SOFAS scores (all *p* > 0.06) (Supplementary Table 1), except for head motion which was higher in the excluded group (*p* < 0.001).

### Computing degree centrality maps

To quantify the whole-brain connectivity of each voxel to the rest of the brain, we employed the metric of degree centrality which was first introduced by^70^. The number of connections each voxel has with every other voxel in the brain above a certain threshold (*r* = 0.25 in this case), determines the degree of centrality (DC) of that voxel. We restricted our voxel-wise analyses of DC to a predefined gray matter mask to control for correlation estimates with non-gray matter voxels. This gray matter tissue probability template, released as a part of the tissue priors in SPM12, was resliced using the “Utilities” function provided by the DPARSFA software to match the dimensions (104 × 104 × 63) of the acquired functional images. The gray matter mask was warped into individual space using DARTEL information to compute DC maps in the “native” space for each subject.

Individual voxel-wise network centrality maps were generated within the study gray matter mask by subjecting the preprocessed functional runs to a time-series correlation analysis conducted for all voxels over the whole brain. The time-series of each voxel within the gray matter mask was correlated with the time-series of every other voxel, which resulted in a time-series correlation matrix. A correlation threshold of *r* > 0.25^63,65,70,71^ was used to generate an undirected adjacency matrix. We computed both binarized DC (bDC—the number of edges in the binarized adjacency matrix, thresholded at *r* = 0.25) and the weighted DC (wDC—the sum of the weights of all pairwise connections for each voxel)^71^. The individual-level voxel-wise bDC and wDC maps were converted into respective *z*-score maps by subtracting the mean DC across the entire brain and dividing by the standard deviation of the whole-brain DC^65,71^, for the individual subject. The resulting maps were then registered into MNI space with dimensions of isotropic 3 mm^3^ voxels using the transformation information acquired from DARTEL. A smoothing kernel of 4 mm full-width at half-maximum was applied after registration to enable parametric mapping. See Supplementary Note 2 for a review on the influence of thresholding procedures used in this study and Supplementary Fig. 2 for the stability of results irrespective of the z-normalization procedure.

As the one-sample and group comparison contrasts for both wDC and bDC were very similar to each other, we only report bDC results in the following section. The wDC maps for primary analysis (HC vs. FEP comparison) are shown in Supplementary Results 1).

### Statistical analysis

#### Group contrasts

We were primarily interested in two contrasts. First, to locate brain regions with aberrant connectivity in relation to FEP, we compared all FEP subjects with control subjects. Second, to locate brain regions with aberrant connectivity in relation to CD, we compared high P2 with low P2 subjects. We estimated the effect sizes of CD-related changes in the eigenvariate of the identified clusters by comparing them against healthy controls.

#### Functional connectivity maps

In order to interpret the specific region-to-region functional connections affected by aberrant bDC, we used a seed-based functional connectivity approach (6 mm sphere around the center of mass of the cluster) and studied the relationship between the time series of BOLD signals of the seed and the rest of the brain within the FEP group.

#### Correction for multiple testing

For all statistical analyses in this study, the threshold for significance was set after correction for multiple testing using a familywise error correction procedure^72^. A conservative cluster inclusion threshold of *p* < 0.001 was used to define the clusters^73^, with a cluster-level significance set at family-wise error (FWE) corrected *p* < 0.05. Given the limitations of cluster wise procedures when making localized spatial inferences, we primarily interpret the location of the center of mass of the identified clusters and not the individual voxel peaks. The nomenclature used in AAL atlas^74^ was used to label the significant clusters. Gender adjustment was used for group contrasts where gender distribution was different (all patients vs. HC) but not for other contrasts.

#### The specificity of findings to P2 scores

Firstly, to demonstrate that the binary split based on a cut-off value does not spuriously result in apparent group differences, we used Spearman’s correlation to relate the eigenvariate of the cluster showing bDC changes in high vs. low symptom severity comparisons to the individual symptom score (see Supplementary Fig. 1 for distribution of P2 scores). Next, to investigate if the findings are specific to P2 as a symptom and no other symptoms of psychosis, we first compared the low and high P2 groups on the distributions of all individual symptom scores (namely, N6—the equivalent of negative FTD, G5, positive symptoms (P1, P3, G9), as well as negative symptoms (N1, N4) of PANSS-8). We also investigated differences in the DSST scores, SOFAS, BNSS between the low P2 and high P2 groups. For the clinical features that differed between the two P2-based groups, we repeated the low vs. high severity between groups analysis of bDC maps. We also undertook a regression analysis, where all symptom scores were entered as predictors and the bDC of the observed cluster as independent variable. All statistical tests were two-sided.

### Reporting summary

Further information on research design is available in the Nature Research Reporting Summary linked to this article.

## Supplementary information

Unmarked supplemental materials

reporting summary

## Data Availability

The data that support the findings of this study are available from the corresponding author upon reasonable request.
