# Supplementary material for: Conceptual disorganization and redistribution of resting-state cortical hubs in untreated first-episode psychosis: A 7T study
Source: NPJ Schizophr. 2021 Jan 26;7:4. doi: 10.1038/s41537-020-00130-3 (PMC7838254; doi:10.1038/s41537-020-00130-3)
Supplement: Supplementary file 1 — Unmarked supplemental materials [file 41537_2020_130_MOESM1_ESM.pdf]

Table of Contents

S1. Influence of assessment procedure 2
S2. Distribution of PANSS P2 scores 3
S3. Demographics of the excluded sample 4
S4. Influence of thresholding parameters.. 5
S5. Weighted degree centrality (wDC) maps 6
S6. Differences between patients and controls for the combined sample 7
S7. Neurosynth meta-analyses.. 8
S8. Effect size of changes in centrality of STG/INS cluster and mSPL cluster 10
S9. Comparison of non-z-score-normalized binarized degree centrality maps across groups..11

Supplementary note 1: Influence of assessment procedure

In the current study, disorganisation among patients was first noted by the referring psychiatrist (names listed in acknowledgment) and confirmed through a clinical interview and rated by one of the 2 research psychiatrists (Kara Dempster & Lena Palaniyappan). Disorganization and the related construct of FTD are both multidimensional constructs, with variations along the axes of subjectivity, positive-negative speech productivity, as well as illness stage related (i.e. acute vs chronic) differences. As recommended by recent comprehensive reviews in this field [1-3], we assessed both a positive (P2 of PANSS) and a negative (N6 of PANSS) feature of FTD. In addition, we also corroborated the presence of disorganization using complementary clinical definitions (YMRS scale items 6 and 7). Nevertheless, our results cannot be taken to represent the neural basis of FTD as such, as we lacked instruments that comprehensively quantify the various aspects of FTD [2, 4].

Supplementary figure 1: Distribution of PANSS P2 scores

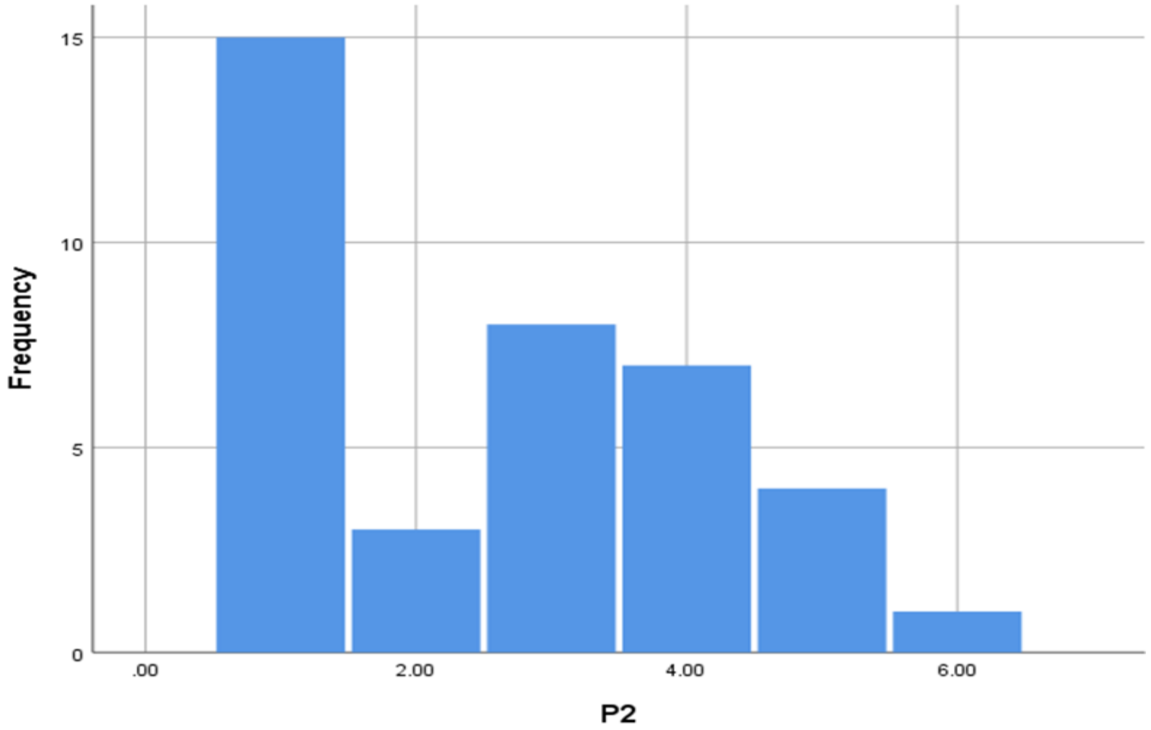

P2- PANSS Positive item 2 – Conceptual Disorganisation

Supplementary table 1: Demographics of the excluded sample

Measure	High P2 (n=4) Mean (SD)	Low P2 (n=9) Mean (SD)	Group statistics
Age (years)	23.02 (3.23)	22.41 (4.65)	T = 0.30; p = 0.77
Gender (M/F) ^	2 / 2	8 / 1	χ2 (1, N = 13) = 2.359; p = 0.125
Exposure to antipsychotic medication (days)	n.a.	n.a.	-
Duration of untreated illness (days)	212.5 (147)	551 (787.85)	T = 0.83; p = 0.42
Total DDD of antipsychotics	n.a.	n.a.	-
Diagnosis (SCZ / Other psychoses) ^	6 / 3	1 / 1‡	χ2 (1, N = 11) = 0.196; p = 0.658
National Statistics Socio-Economic Classification score	3.11 (1.45)	2.33 (1.53)	T = 0.80; p = 0.45
Conceptual disorganization (P2) †	4.56 (0.527)	2.00 (1.16)	U = 0.000, p = 0.003
Lack of spontaneity & flow of conversation (N6) †	1.56 (0.882)	1.75 (0.957)	U = 15.500, p = 0.710
Total positive component (P1 + P3 + G9)	13.22 (2.54)	10.75 (0.96)	U = 8.000, p = 0.148
Total negative component (N1 + N4)	3.56 (2.83)	5.00 (2.16)	U = 8.000, p = 0.148
Speech rate & amount (YMRS 6) †	1.21 (1.72)	0 (0)	U = 10.000, p = 0.260
Language / Thought disorder (YMRS 7) †	2.79 (1.27)	0.73 (1.85)	U = 5.000, p = 0.05
SOFAS	35.22 (10.39)	46.50 (11.21)	T = 1.767; p = 0.105
DSST (Mean)	46.39 (9.97)	57.25 (2.90)	T = 2.093; p = 0.06

^ chi-square test; †Mann Whitney U-test; S.D. = Standard deviation; ‡data unavailable for other 2 subjects; DDD = Defined daily dose of antipsychotics; High P2: Patients with high CD; Low P2: Patients with low CD

Supplementary note 2: Influence of thresholding parameters

While computing topological properties such as centrality based on graph theory to study brain connectomes, a limited number of brain regions are usually selected as nodes of interest, and sparse connectivity matrices are obtained to delineate the networks of interest. The 2 commonly used approaches to generate sparse matrices are (1) the use of an absolute edge-defining threshold (e.g. edges with functional connectivity value above 0.25 are retained as in [5]) or (2) using a fixed edge density threshold (e.g. 30% of all possible edges are retained in the matrices). The use of an absolute edge defining threshold can result in various sparsity values across different individuals (or groups). As several topological metrics depend on the degree of sparsity [6], the resulting differences may indeed be due to variations in the number of edges rather than true differences in the network topology. Proportional thresholding using several values of fixed edge density obviates this problem (e.g. [7]). But when using this approach, if systematic differences in the global strength of connectivity exist between two groups of interest, this can result in noisy and spurious edges with low connectional strength being included in one group and not in the other. Again, this could lead to an apparent group difference in topological metrics, with a shift towards randomness in the group with weaker overall connectivity [8, 9]. Our primary interest was not in deriving the topological architecture of functional connectivity. Instead, we were focused on deriving a single score for each voxel that best represented the overall (voxelwise) functional connectivity required to characterize hubs. As a result, we used Buckner's approach [5] in this study. Our previous use of this approach resulted in highly reproducible hubs across different brain states (rest, 0,1, and 2 back task performance) [10]. For the same reasons of continuity with the prior work as well as reproducibility of hubs in patient datasets, we did not employ eigenvector centrality, a measure that is computationally more efficient and does not require a binarizing threshold [11,12].

Supplementary results 1: Weighted degree centrality (wDC) maps

We found a similar reduction in weighted degree centrality in the STG/Insula cluster with same coordinates (x = 48, y = -9, z = 3) for the peak of the cluster and same peak statistics (T = 5, p = 0.001) as observed for the two sample t-test results of the binarized degree centrality maps in our sample.

Cluster information					Peak voxel statistics	
Brain regions included in cluster (AAL labels)	Cluster size	Peak MNI coordinates			T- value	p-value (cFWE corrected)
		x	y	z		
FEP < HC						
STG/Insula cluster	201	48	-9	3	5.00	0.001
Temporal_Sup_R	88					
Insula_R	56					
Heschl_R	16					
FEP > HC						
Not significant						

cFWE = cluster FWE; MNI = Montreal Neurological Institute; FEP = First episode psychosis; HC = Healthy controls

Brain regions showing wDC alterations in the FEP group (n = 38) compared to the HC group (n = 31).

Supplementary table 2: Differences between patients and controls for the combined sample

Measure	Group		Statistics
	First Episode Psychosis	Healthy Controls	
	Mean (S.D.)	Mean (S.D.)	
Number of subjects	38	31	
Age (years)	22.47 (4.50)	21.61 (3.26)	T = -0.886; p = 0.379
Gender (M/F)	32 / 6	19 / 12	χ2 (1, N = 69) = 4.652; p = 0.03
Framewise Displacement	0.18 (0.05)	0.15 (0.04)	T = -2.502; p = 0.015*
Total intracranial volume	1540.78 (136.53)	1554.51 (148.48)	T = 0.400; p = 0.691
Total gray matter volume	693.24 (62.55)	706.18 (76.09)	T = 0.775; p = 0.441

Supplementary discussion 1: Neurosynth meta-analyses

For the STG/Insula cluster

After obtaining functional connectivity maps of the STG/Insula cluster (showing reduced hubness in the patients vs. healthy controls) with the rest of the brain for healthy controls, we wanted to assess the comparability of our results with those observed in previous literature. We searched the Neurosynth online database (www.neurosynth.org) for meta-analytic maps of functional connectivity of the cluster belonging to that coordinate (x = 48, y = -9, z = 3). Our observations of the brain regions which were functionally connected to the STG/Insula cluster were similar to findings from the existing literature, confirming the comparability of our study to the literature (Figures 1 & 2).

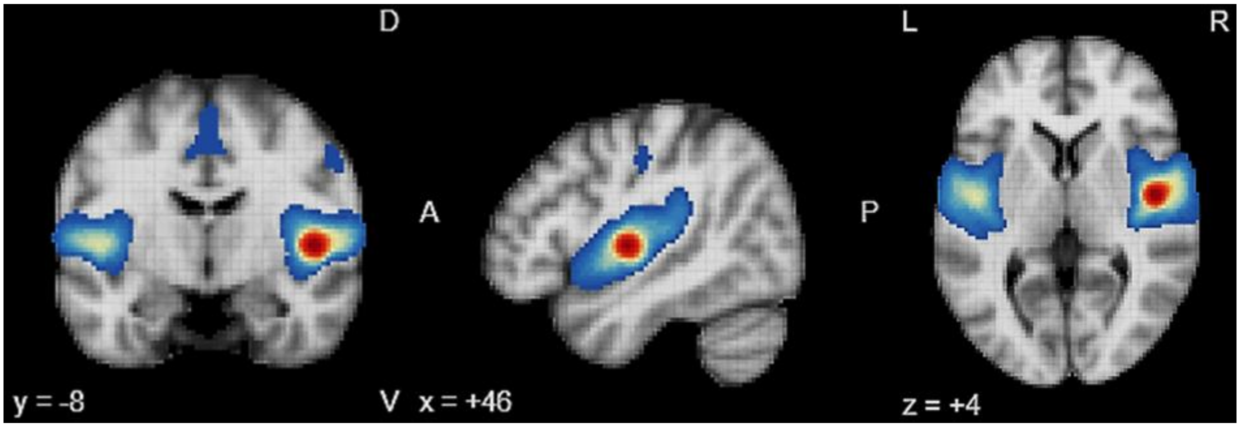

Meta-analytic results (obtained from the Neurosynth database) of functional connectivity associations between the STG/Insula cluster (x = 48, y = -9, z = 3) and the rest of the brain

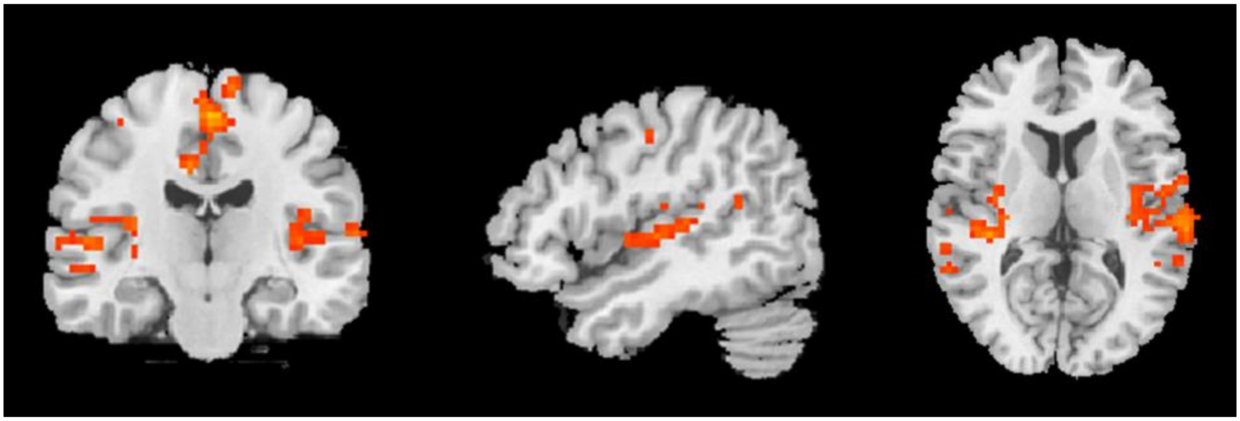

Functional connectivity associations between the STG/Insula cluster (x = 48, y = -9, z = 3) and the rest of the brain

For the medial superior parietal cluster:

We performed a similar comparability check for the mSPL cluster showing increased hubness in the patient group with high disorganization vs. the patient group showing low disorganization. Results from the meta-analytic maps of functional connectivity of the cluster belonging to that coordinate (x = -12, y = -30, z = 69) showed similarities to our findings of functional connectivity (Figures 3 & 4).

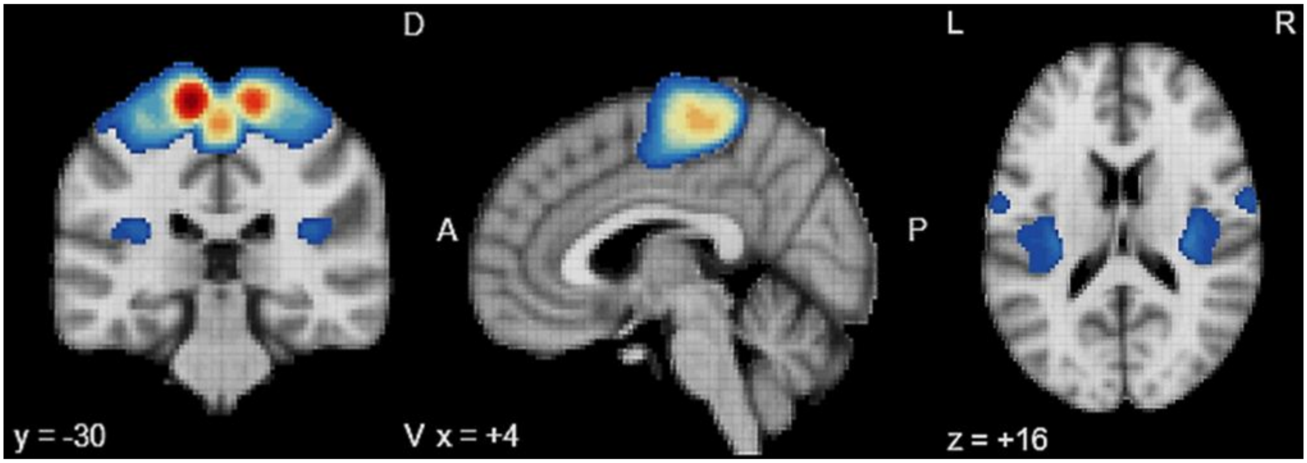

Meta-analytic results (obtained from the Neurosynth database) of functional connectivity associations between the medial superior parietal cluster (x = =12, y = -30, z = 69) and the rest of the brain.

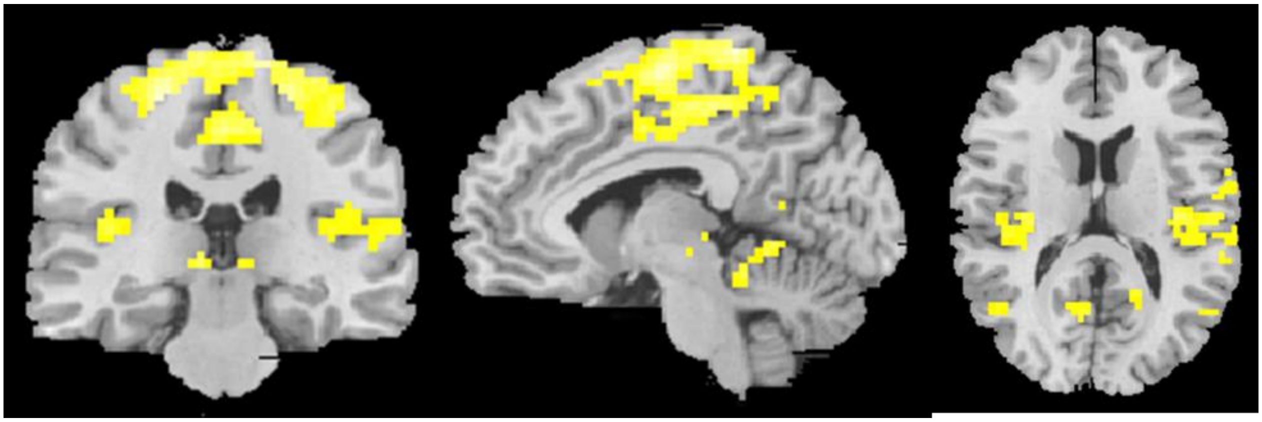

Functional connectivity associations between the medial superior parietal cluster (x = =12, y = -30, z = 69) and the rest of the brain.

Supplementary results 2: Effect size of changes in centrality of STG/INS cluster and mSPL cluster

To understand the gradient of changes in the 2 clusters observed from patients vs. healthy controls (STG/INS) and high vs. low P2 groups (mSPL), we estimated the effect sizes (Cohen's d) of the group differences. These results demonstrate the reduction of STG/INS centrality across all patients (Cohen's d of 0.98 to 1.26), but only those with high P2 show an equal sized effect of increased mSPL centrality when compared to healthy controls.

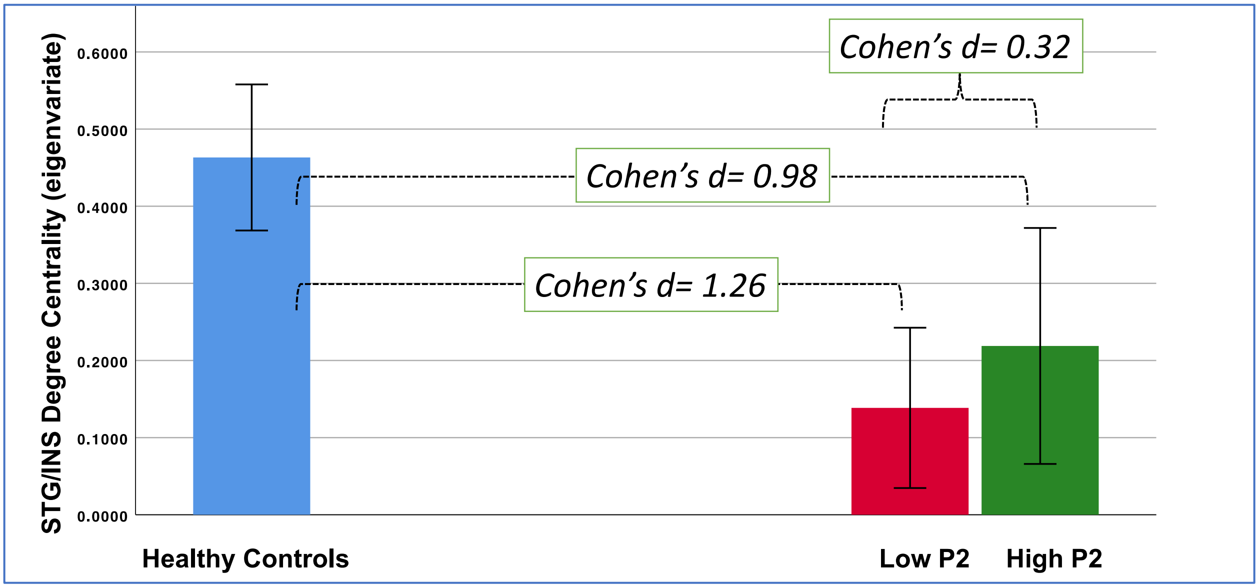

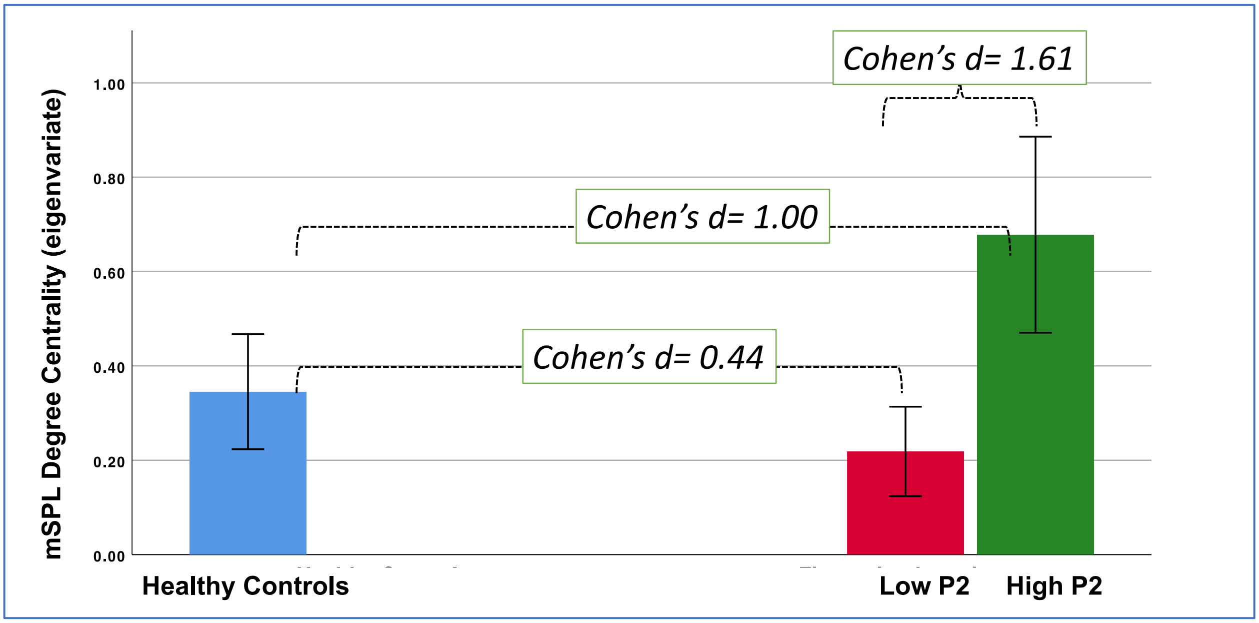

Supplementary figure 2: Comparison of non-z-score-normalized binarized degree centrality maps across groups

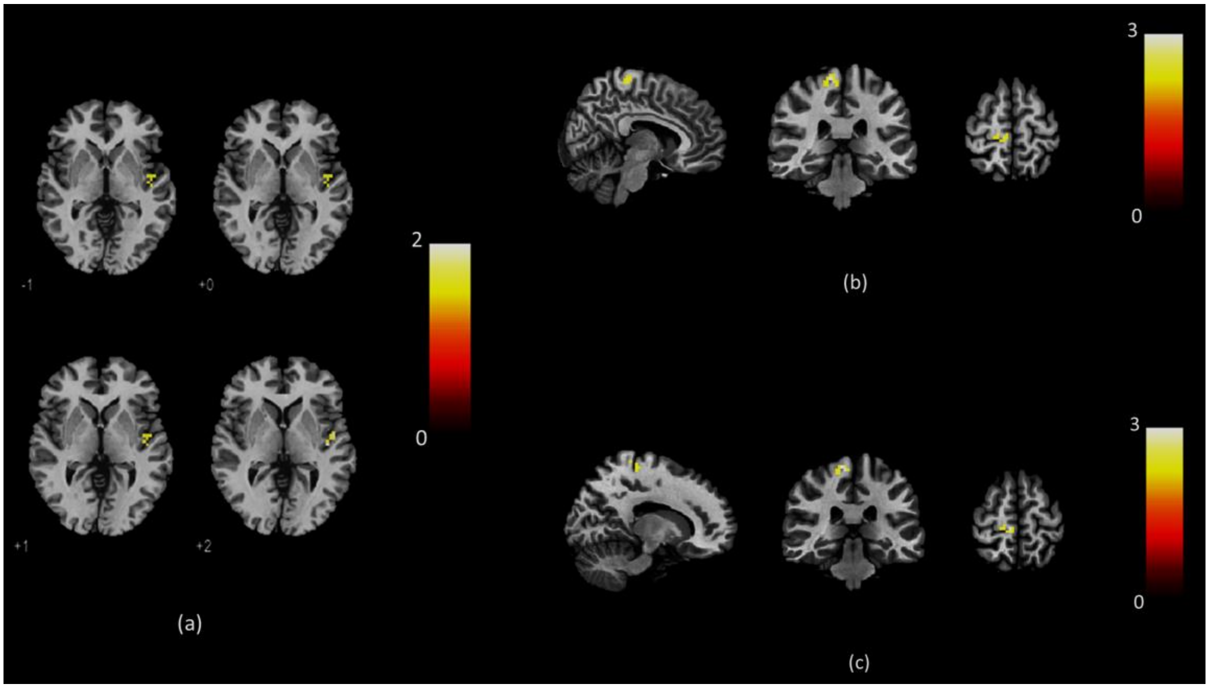

Brain regions showing differences in non-z-score-normalized degree centrality for (a) FEP < HC, (b) High P2 > Low P2, and (c) High P2 > HC contrasts.

To ascertain that the differences observed on comparing z-score-normalized degree centrality maps were representative of true pathology, rather than a result of the normalization process, we repeated the group comparison contrasts on non-z-score-normalized degree centrality maps between the HC and FEP (including subtypes based on P2 scores) groups (Figure S9), constrained to STG/INS and mSPL regions. We observed reduced centrality of STG/INS in FEP compared to HC, and increased mSPL centrality in high P2 compared to low P2 and HC, irrespective of whether normalization was employed or not.

References:

1. Cavelti, M., Kircher, T., Nagels, A., Strik, W. & Homan, P. Is formal thought disorder in schizophrenia related to structural and functional aberrations in the language network? A systematic review of neuroimaging findings. Schizophrenia Research 2018; 199: 2–16.
2. Kircher, T., Bröhl, H., Meier, F. & Engelen, J. Formal thought disorders: from phenomenology to neurobiology. The Lancet Psychiatry 2018; 5: 515–526.

3. Sumner, P. J., Bell, I. H. & Rossell, S. L. A systematic review of task-based functional neuroimaging studies investigating language, semantic and executive processes in thought disorder. Neuroscience and Biobehavioral Reviews 2018; 94: 59–75.
4. Strik, W. et al. The Bern psychopathology scale for the assessment of system-specific psychotic symptoms. Neuropsychobiology 2010; 61: 197–209.
5. Buckner, R. L. et al. Cortical Hubs Revealed by Intrinsic Functional Connectivity: Mapping, Assessment of Stability, and Relation to Alzheimer's Disease. Journal of Neuroscience 2009; 29: 1860–1873.
6. Tewarie, P. et al. Functional brain network analysis using minimum spanning trees in Multiple Sclerosis: An MEG source-space study. NeuroImage 2014; 88: 308–318.
7. Bassett, D. S. et al. Hierarchical Organization of Human Cortical Networks in Health and Schizophrenia. Journal of Neuroscience 2008; 28: 9239–9248.
8. van den Heuvel, M. P. et al. Proportional thresholding in resting-state fMRI functional connectivity networks and consequences for patient-control connectome studies: Issues and recommendations. NeuroImage 2017; 152: 437–449.
9. Váša, F. et al. Adolescent tuning of association cortex in human structural brain networks. Cerebral Cortex 2018;28: 281–294.
10. Palaniyappan, L. & Liddle, P. F. Diagnostic discontinuity in psychosis: A combined study of cortical gyrification and functional connectivity. Schizophrenia Bulletin 40, 675–684 (2014).
11. Skåtun, K. C. *et al.* Global brain connectivity alterations in patients with schizophrenia and bipolar spectrum disorders. *J Psychiatry Neurosci* **41**, 331–341 (2016).
12. Lohmann, G. *et al.* Eigenvector Centrality Mapping for Analyzing Connectivity Patterns in fMRI Data of the Human Brain. *PLOS ONE* **5**, e10232 (2010).
